# Two-photon 3D imaging of optically stimulated neural activity at 100 Hz

**DOI:** 10.1038/s41377-026-02395-2

**Published:** 2026-07-03

**Authors:** Dongli Xu, Fuu-Jiun Hwang, Jun B. Ding, Leilei Peng

**Affiliations:** 1 Wyant College of Optical Sciences, University of Arizona, Tucson, AZ 85721 USA; 2 Department of Neurosurgery, Stanford University, Stanford, CA 94305 USA; 3 Department of Neurology and Neurological Sciences, Stanford University, Stanford, CA 94305 USA; 4 Wu-Tsai Neuroscience Institute, Stanford University, Stanford, CA 94305 USA

**Keywords:** Imaging and sensing, Biophotonics

## Abstract

Understanding how neurons integrate synaptic inputs requires imaging techniques capable of capturing rapid, three-dimensional dendritic events. These processes occur on millisecond timescales and submicron spatial scales, exceeding the speed of conventional two-photon microscopy (2PM). We developed dual-view Bessel two-photon projection microscopy (dv-B2PM), a high-speed volumetric imaging approach that achieves 100 Hz whole-volume acquisition with synaptic-level resolution. dv-B2PM simultaneously records two orthogonal projections of the same 3D volume, preserving spatial information while minimizing ambiguity from structural overlap. Combining dv-B2PM with two-photon glutamate uncaging, we visualized 3D Ca²⁺ dynamics in neurons following localized stimulation. Multi-timescale analysis revealed dendrite-to-soma Ca²⁺ signal propagation, back propagated Ca²⁺ signal from the soma, and multi-frequency (5–40 Hz) Ca²⁺ transients activated along apical dendrites at speeds from ten of microns per second to millimeters per seconds. These findings demonstrate dv-B2PM as a powerful tool for direct visualization of 3D calcium dynamics associated with dendritic integration across extended neuronal structures, bridging the gap between optical imaging and the dynamic biophysics of neuronal integration.

## Abstract (web summary)

Dual-view Bessel two-photon projection microscopy imaged 3D calcium signals in brain tissue at 100 Hz, revealing 5–40 Hz neuronal calcium transients traveling bi-directionally along the dendrite following two-photon glutamate uncaging.

## Graphical abstract

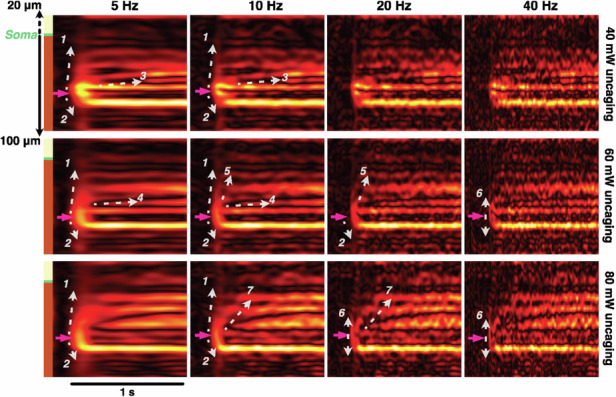

## Introduction

Understanding how neurons perform computations requires observation of how they integrate synaptic inputs across space and time. Modern neural imaging has become indispensable for this purpose, as it allows researchers to visualize electrical and biochemical signaling within living neural circuits. Among these processes, dendritic integration plays a central role: excitatory and inhibitory synaptic inputs arriving on dendritic arbors are combined through complex, nonlinear mechanisms that determine how neurons encode and transmit information. These computations involve a complex calcium (Ca²⁺) toolkit^1^ that generates rich spatial-temporal activity, from fast, localized synaptic Ca²⁺ transients to back-propagating action potentials (bAPs) and propagating calcium waves^2^ across extended dendritic branches^3^. Capturing such events requires an imaging system capable of resolving sub-micron spatial features and millisecond-level temporal dynamics within large volume of three-dimensional (3D) neuronal structures.

Two-photon microscopy (2PM) has long served as a cornerstone of neural imaging because of its deep tissue penetration, optically confined excitation, low background and minimal photodamage, thereby enabling higher clarity imaging within three-dimensional (3D) neural structures. When combined with two-photon glutamate uncaging^4^, 2PM enables precise stimulation of individual synapses and high-resolution monitoring of neuronal responses in intact brain tissues^4,5^. These features have made 2PM indispensable for studying how synaptic inputs are integrated along dendrites to generate local dendritic spikes and plateau potentials that contribute to neuronal output. However, the conventional 2PM technique relies on point scanning, which inherently limits its imaging speed. This constraint makes it difficult to capture rapid and volumetric neural events associated with dendritic integration, which occur on the millisecond timescale. Compared with one-photon microscopy or electrophysiological techniques such as patch clamp recording, 2PM offers superior spatial resolution and low background imaging in deep tissues, but its slower acquisition speed remains a key bottleneck for studying fast 3D computations occurring within neurons. 2PM imaging speed can be increased to kHz frame per second (FPS) range using customized scanning speed multiplier^6^ but the signal-to-noise ratio drops because of the much shorter integration time. Parallel excitation^7,8^ can also acquire multiple frames simultaneously, but the multiplication factor is limited by the laser power safety limit in live biological sample. Moreover, even a kHz frame rate would only translate to only a few tens of volume per second because most 3D tissue required close to tens of layers of 3D scan.

Live neural activity poses additional challenges in high-speed imaging. Weak signals in such applications often force the imaging instrument to operate at a lower speed to improve the signal to noise ratio (SNR). For example, a high speed one-photon excitation light-sheet instrument achieved 300 volumes per second (VPS) in structural imaging, but dropped to 6 VPS when imaging somatic activities^9^. This challenge is much harder in 2PM of fine neural structures, such as spines and dendrites, because signals from these fine neural structures, combined with lower excitation rate of 2PM, are substantially weaker than one-photon signals from soma.

Instead of pushing for higher instrument speed, several groups have focused on selectively detecting image information to increase the effective 3D acquisition rate. Selected area imaging, achieved through random accessing^10–12^, targeted path scanning^13^ or light pattern modulation^5^, accelerates imaging acquisition by focusing only on regions of interest. Projection imaging techniques compress 3D structures into two-dimensional (2D) projections, allowing video-rate detection of the whole volume through 2D imaging^14–16^. However, these high-speed 2PM strategies inevitably sacrifice some information: selected area imaging neglects information beyond the chosen observation area, and 2D projection imaging introduces ambiguities on overlapping structures because of the loss of depth information. For studies of dendritic computation, where signals may propagate in 3D along branching dendrites, these limitations restrict the ability to reconstruct how plateau potentials and Ca^2+^ transients evolve across the neuronal arbor.

Here, we introduce a dual-view Bessel two-photon projection microscopy (dv-B2PM) technique that addresses these challenges by enabling whole-volumetric imaging at 100 VPS with synaptic-level resolution. dv-B2PM uses projection imaging to achieve 100 VPS while preserving the depth information by simultaneously capturing two orthogonal projection images of the same 3D volume. Its volumetric refreshing rate is comparable to that of a 14 kHz-frame-rate 2PM, but it operates at a 100 FPS imaging-acquisition rate, which provides a lower noise floor and better sensitivity to weak signals. The dual-view approach minimizes structural ambiguities that often arise in projection imaging, allowing us to follow the propagation of dendritic signals in 3D space at a unprecedented speed.

We further combined dv-B2PM technique with two-photon (2P) glutamate uncaging to study 3D Ca^2+^ dynamics in neurons after localized stimulation. This combination allowed us to observe multi-timescale spatiotemporal Ca^2+^ activities extending from the apical dendrite to the soma. Long timescale spatiotemporal signal model fitting revealed that Ca^2+^ waves were driven by the dendrite-to-soma signal propagation and by back-propagated action potentials (bAPs) from the soma. Short timescale 2D wavelet analysis identified regional fast transient Ca^2+^ signals around 5–40 Hz that originated from the uncaging site and propagated along apical dendrites at velocities ranging to from tens of microns per second to millimeters per second. These observations highlight dv-B2PM’s ability to resolve both local dendritic integration and long-range signal spreading within neurons.

**Fig. 1:**
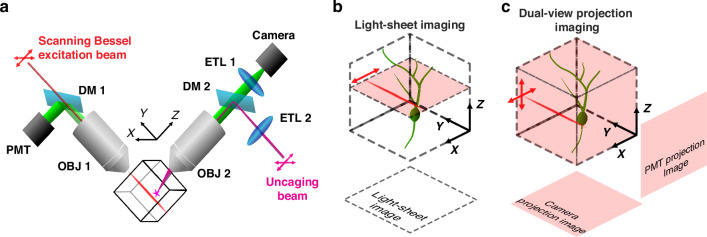
Schematic of dual-view Bessel 2P projection microscopy (dv-B2PM) with 2P uncaging. **a** A schematic of dv-B2PM. The dv-B2PM setup is based on an upright light-sheet microscope with two 90-degree configured objective lenses. The Bessel excitation beam scanned across the imaged volume. X: the fast-scanning direction. Y: the excitation beam propagating direction. Z: the low-speed scanning direction. **b** In the light-sheet imaging mode, each camera exposure captures a single X–Y optical section of the volume. **c** In the dual-view projection imaging mode, a PMT and a camera capture two orthogonal projections of the whole volume. A second laser beam coupled into the camera path to perform two-photon glutamate uncaging. The uncaging beam can be independently positioned laterally by a close loop 2D laser scanner. Two independent tuned electrically tunable lenses (ETL1 and ETL2) are used to control the z-focusing of the camera imaging and the uncaging beam respectively. PMT: photon multiplier tube. DM: dichroic mirror. OBJ: objective lens

The dv-B2PM optical design is based on a two-photon Bessel light-sheet microscope^17^ with minor hardware modifications (Fig. 1a). The system employs a Bessel beam to raster scan across the imaged volume, forming an extended, thin 2P light-sheet that supports high-resolution 3D imaging over a large field of view^18–20^. The microscope uses a costume designed upright configuration with two 90-degree-oriented objectives. In its standard mode (Fig. 1b), one objective lens (OBJ1) delivers the scanning Bessel 2P excitation beam while the other objective lens (OBJ2) collects fluorescence to reconstruct 3D volumes through multiple exposures^17^. To enable dv-B2PM and 2P uncaging, we added a photomultiplier tube (PMT) behind OBJ1 and introduced an independently controlled uncaging laser through the OBJ2. Both objective lenses thus serve dual purposes: OBJ1 delivers the Bessel 2P excitation laser beam and collects fluorescence signals to the PMT, whereas OBJ2 delivers the 2P uncaging laser and collects emitted fluorescence.

The key innovation of the dv-B2PM lies in its dual-view acquisition mode (Fig. 1c). Instead of capturing sequential imaging stacks, the system simultaneously acquires two synchronized projection images from orthogonal angles during each high-speed volume scan. One projection image is recorded by the PMT, and the other by a camera whose focal plane dynamically tracks the Bessel beam. This approach compresses volumetric information into single exposures, eliminating delays caused by mechanical focusing and camera readout, while the two orthogonal views preserve 3D spatial information. Together, the projection image pair allows 3D localization of dendritic signals within the imaging volume. Using dv-B2PM, we achieved 100 volumes per second (VPS) imaging over a 120 × 80 × 42 µm^3^ volume field of view with 0.5 × 0.5 × 0.75 µm^3^ optical voxel resolution and a digital voxel size of at 0.3 × 0.3 × 0.3 µm^3^, enabling direct visualization of dendritic integration and plateau potential propagation with both high spatial and temporal fidelity.

## Results

### Two-photon glutamate uncaging near a single dendrite

The combination of fast volumetric dv-B2PM imaging and precise 2P glutamate uncaging enables visualization of dendritic Ca^2+^ activity under controlled stimulation, allowing observation of signals originating from the apical dendrite near the uncaging point and being relayed toward the soma. In GCaMP8m-labeled layer 5/6 of the motor cortex (M1) brain slices, we selected uncaging locations near dendritic spines using the 3D light sheet image as a guide. Ca^2+^ activity was captured during repeated uncaging events delivered near a single dendrite.

We observed two distinct spatial-temporal Ca^2+^ activity modes following 2P glutamate uncaging adjacent to a single spine. The first mode, corresponding to sub-threshold stimulation, produced Ca^2+^ signals restricted to a short dendritic segment surrounding the uncaging site (Fig. S1a). The associated Ca^2+^ transients exhibited a single, brief peak (Fig. S1b, c). The second mode, reflecting suprathreshold stimulation, resulted in Ca^2+^ activity spreading to the soma and additional dendritic branches (Fig. S2a). In this suprathreshold stimulation condition, Ca^2+^ signals frequently displayed a slow rising secondary surge following the initial peak (Fig. S2b, c), and the decay phase was substantially prolonged. In some locations, long-lasting plateau signals emerged. These suprathreshold Ca^2+^ dynamics suggest that somatic activity may contribute to secondary surges and plateau phases observed in dendrites.

**Fig. 2:**
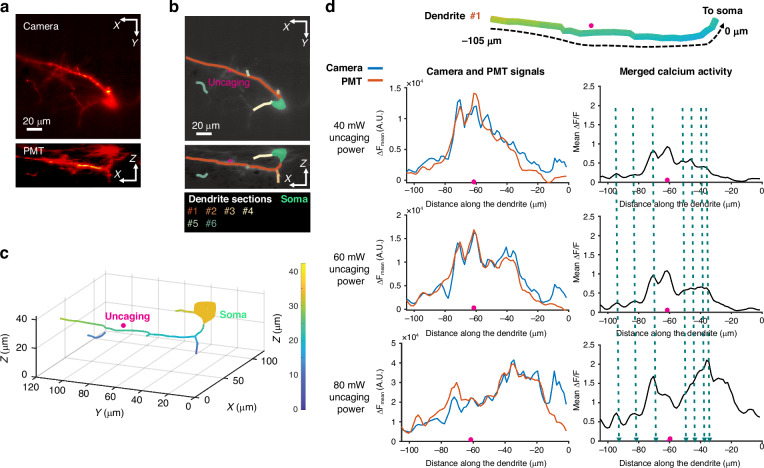
Dual-view Bessel 2P projection imaging of calcium responses evoked by 2P glutamate uncaging. **a** Dual view calcium activity images of a neuron after uncaging, captured by the camera and the PMT of dual-view Bessel projection microscope at 100 Hz. The images are an average of calcium signal increase (ΔF) during the first 5 s after uncaging onset using an 80 mW uncaging beam power. The imaged volume was 120 μm × 80 μm × 42 μm. **b** Traced 3D structure of the neuron in the dual view projection images. Major structures were assigned with different colors. The uncaging location is marked (magenta dot) in both projection images. **c** Reconstructed 3D structures of the neuron. Depth of dendrites are indicated by the color map. **d** Time-averaged calcium signal increases (ΔF) along dendrite #1 within the first 5 s after uncaging. Left column: Peak ΔF along the dendrite overserved by the camera and the PMT are consistent with each other under all three stimulation levels. Therefore, ΔF observed by the camera and PMT are merged into a signal value in further analysis. Right column: ΔF value varied depending on the power of the uncaging beam but locations of local maximums of ΔF (hot spots, indicated by doted lines) are consistent across all uncaging levels

To investigate the transition between sub- and supra-threshold Ca^2+^ activity modes and their dependence on stimulation strengths, we applied repeated high-frequency uncaging stimulation near the apical dendrite of a neuron while varying the uncaging beam power. dv-B2PM imaging at 100 VPS captured Ca^2+^ signals across the extended structure from the apical dendrite to the soma and basal dendrites (Fig. 2). By adjusting the uncaging laser power, while maintaining the uncaging stimulation duration and duty cycle constant, we could reliably evoke both sub- and supra-threshold activity patterns within the same neuron.

The uncaging simulation site was positioned about 1 μm away from a distal section of the apical dendrite (dendrite #1, Fig. 2). Under weaker uncaging powers (40 mW and 60 mW), Ca^2+^ activity remained confined to dendrite#1 between the uncaging site and the soma, with the strength of Ca^2+^ signals decaying before reaching the soma. In contrast, high-power uncaging stimulation produced strong Ca^2+^ signals throughout major dendritic structures and the soma, enabling 3D tracing and reconstruction of the neuron (Fig. 2b, c). Notably, the neuron possessed major dendritic segments oriented perpendicular to one of the projection planes, which would be obscured using single view 2D projection imaging. The dual projection dv-B2PM images allowed unambiguous measurement of Ca^2+^ signals in these orthogonal segments.

### Long time-scale spatial-temporal analysis of Ca^2+^ signals

**Fig. 3:**
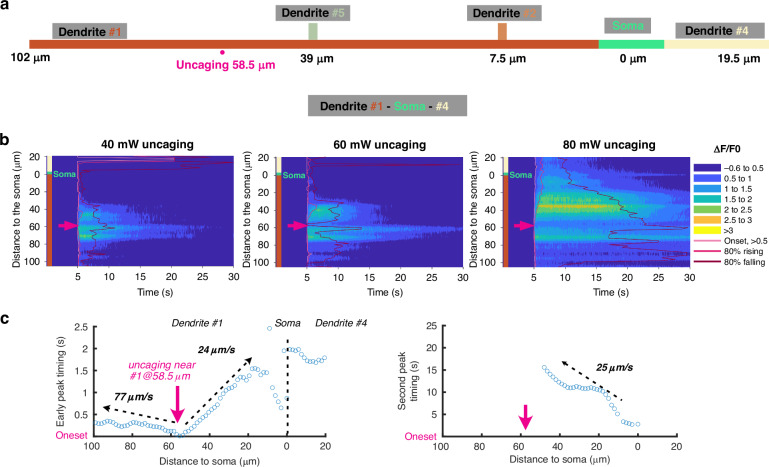
Spatiotemporal calcium activities in the neuron under increasing single-point uncaging stimulation. **a** Diagram of dendrite branches, lengths of major sections and the location of uncaging. Lengths are marked by distances from the soma. The dendrite section closest to the uncaging point located at 58.5 µm on dendrite #1. **b** Calcium activities spatiotemporal heat map of the signal path from apical dendrite #1, which is the targeted by uncaging, to soma and dendrite #4. Magenta markers indicate the nearest dendrite location to the uncaging point or uncaging timing at 5 s. The onset timing (ΔF/F_0_ > 0.5), 80% ΔF/F_0_ peak on the rising edge, and 80% ΔF/F_0_ peak on the falling edge are marked by red lines. See Fig. S3 for calcium time traces at selected locations along the dendrite #1-to-soma-to-dendrite #4 path. **c** Long timescale analysis results. The analysis fit signals stimulated by 80 mW uncaging beam power with a double-peak model (Fig. S4). At locations where there are not distinct secondary peaks, the fitting converged to the single peak model and assigned a zero amplitude on the second peak. Left: Timing of the early or single peak. Right: Timing of the second peak. The early peak delays by approximately 24 µm/s toward the soma and 77 µm/s toward the distal end. Second peaks are detectable on dendrite #1 between the soma and the uncaging point. The timing of second peaks is the earliest near the soma and delays toward the uncaging point. The nonlinear delay is in average at a rate of 25 µm/s

Ca^2+^ signals captured with dv-B2PM exhibited rich spatial and temporal information. Spatially, a series of Ca^2+^ activity “hot spots” that had high average calcium appeared along the apical dendrite under both sub- and suprathreshold stimulation. These hot spots, separated approximately 5–15 µm apart, remained fixed in position across all uncaging power levels (Fig. 2d). Temporally, Ca^2+^ signals displayed slow-varying peak-decay dynamics superimposed with fast fluctuation. Figure 3 shows Ca^2+^ signal time traces along the signal path from dendrite #1 to soma and a basal dendrite #2. On the long timescale, Ca^2+^ signals fit either subthreshold or suprathreshold response profiles. After weak uncaging stimulation (40 mW and 60 mW), Ca^2+^ signals in dendrite #1 exhibited a single peak whose amplitude decayed toward the soma. Only small, brief Ca^2+^ level peaks were observed in the soma and dendrite #2, with much smaller amplitude and duration than those near the uncaging site.

In contrast, strong uncaging stimulation (80 mW) elicited prominent slow-rising Ca^2+^ surges in the soma and in dendritic segments directly connected to the soma, indicating a suprathreshold neuronal response. In dendrite #1 near the uncaging point and at the distal end, peak amplitudes did not change substantially, but the decay phase lengthened markedly. Ca^2+^ signals in dendrite regions between the uncaging site and the soma reflected a mixture of two components: an early rising peak resembling the localized dendritic response near the uncaging point, and a slower secondary surge resembling the somatic response (Fig. S3). The coexistence of these two peak types, one matching localized dendritic Ca^2+^ signals and one resembling somatic Ca^2+^ dynamics, strongly suggests that the observed Ca^2+^ signals resulted from both dendritic integration of the initial suprathreshold input signals and back-propagated action potentials (bAPs) originating from the soma^21^.

Peak fitting of long-timescale Ca^2+^ dynamics further supported this interpretation (Fig. 3c). We fit the Ca^2+^ signals with a dual-peak model containing early and late exponentially modified Gaussian components (Fig. S4). Secondary late-arriving peaks were apparent in dendritic regions between the soma and the uncaging site, whereas signals in other regions were adequately modeled with a single peak. From these fits, we extracted the timing of the early peak (or single peak). Near the uncaging site, this early peak occurred within 10 ms of the stimulation, with delays increasing linearly toward both the soma and the distal end at propagation speed of 24 µm/s and 77 µm/s, respectively (Fig. 3c left). These long timescale peaks, although exhibiting relatively show rise and decay kinetics overall, traveled between dendritic Ca^2+^ hot spots and branching points over intervals as short as few tens of milliseconds. dv-B2PM provided the critical 100 Hz speed needed to observe their propagation with high structural resolution.

These delays indicate that the initial rise in Ca^2+^ was driven by localized suprathreshold stimulation, whose downstream effects propagating bidirectionally along the dendrite. In contrast, the late peaks appeared earliest in the soma and were delayed progressively toward the uncaging site at ~25 µm/s (Fig. 3c right), consistent with Ca^2+^ signal associated with bAPs traveling from the soma outward.

The propagation speeds of both peak classes and their slow decay kinetics are consistent with Ca^2+^ waves^3^ reported under synaptic stimulation^22,23^. The observation of increasing stimulation strength promoted broader signal spread and eventually triggered a somatic response is also consistent with prior studies^22,23^. These studies, however, were conducted with low-resolution 2D epifluorescence whole cell imaging at a low imaging speed and could not resolve fast Ca^2+^ transients associated with voltage and ligand-gated channels. In our study, the whole cell was imaged with 3D synaptic resolution and 100 Hz, allowing us to analysis transient signal riding on top of slow Ca^2+^ waves. According to the Nyquist sampling rule, a 100 Hz sampling rate can detect signal components up to 50 Hz.

### Short timescale spatial-temporal analysis of Ca^2+^ transients

**Fig. 4:**
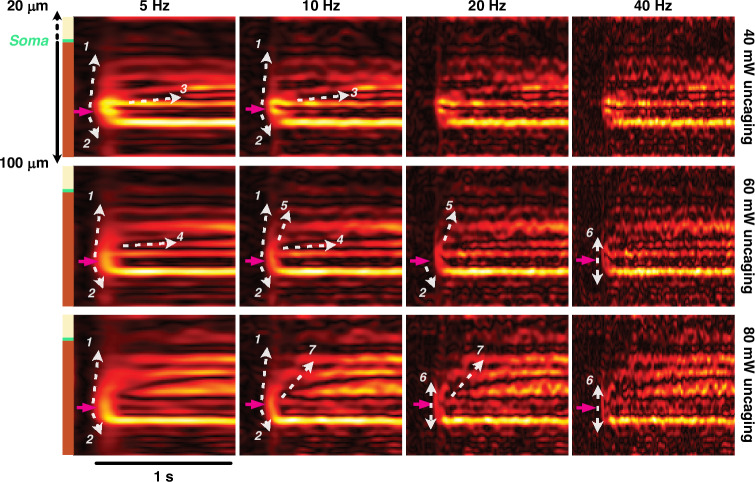
Short timescale spatial-temporal analysis of calcium signal along the signal path. The analysis used 2D wavelet analysis on signals from dendrite #1 to soma and dendrite #4. Four wavelets centered at 5, 10, 20 and 40 Hz were used. The nominal spatial resolution of all wavelets was kept the same at 5 µm. The nearest location to the uncaging point is marked by the magenta arrow. These heat maps are modular amplitudes of 2D continues wavelet analysis of calcium signals. Highlighted area in heat maps indicate transients that persists in space and time. Several traveling traces are visible in these maps. Their spatial temporal characteristics are listed in Table S1

Ca^2+^ transients on shorter timescale were intermixed with stochastic noises. To isolate high levels of transients temporally and spatially, we applied a 2D continuous wavelet analysis transform to the spatiotemporal Ca^2+^ activity maps. The analysis utilized wavelet kernels to target individual peak-to-valley fluctuations at four-time scales. The resulting wavelet maps (Fig. 4) revealed Ca^2+^ transients at 5–40 Hz. In left side edges of all panels, which correspond to calcium baseline signals before stimulations, there were only random isolated reading, indicating the wavelet analysis rejected random noises. Highlighted spatial-temporal areas of transients became visible after the simulation. There were transients remaining at high active levels over time at hot spots (horizontal highlighted areas) and short duration of strong transients moving over space (tilted highlighted areas) along the dendrite #1 → soma → dendrite #4 path.

The most prominent spatial-temporal traces of moving transients (trace #1 and #2 in Fig. 4) originated at the uncaging spatial-temporal point and moved bidirectionally along the dendrite. They are consistent with dendritic propagation of the initial uncaging-evoked dendritic Ca^2+^ signals and visible at all stimulation power levels. Additional traces appeared and disappeared depending on the uncaging power and frequency range. These Ca^2+^ transients’ movements spanned fast, slow, long-range and regional signal transport along the dendrite (Table. S1). For example, a high-frequency burst along the dendrite (trace #6) happened immediately after the stimulation and traveled at a speed beyond the measurement range, suggesting it may reflect voltage-induced Ca^2+^ transients. A long-range-moving Ca^2+^ transient at in 5–10 Hz frequency bands traveled toward soma at approximately 1.5 mm/s immediately after the stimulation (trace #1), indicating rapid long-range signal transport. Multiple 5–20 Hz transients initiated at the stimulation location, traveled regionally at tens to hundreds of microns per second and ended with high levels of transients remaining at hot spots (trace #2–5), suggesting Ca^2+^ hot spots are terminals of regional signal transport and generate persistent local Ca^2+^ transients upon receiving a signal. All spatial-temporal traces showed increasing temporal delay with distance from the uncaging site, indicating that these fast Ca^2+^ fluctuations were initiated by the uncaging stimulation and spread outward from the dendrite origin closest to the uncaging point. Evidence of soma-initiated transients was not observed, even under the suprathreshold stimulation. Similar spatial-temporal patterns were also observed in wavelet analysis of an independent image data set (Fig. S1d). Further experiments—such as simultaneous electrophysiology or pharmacological perturbations—will be required to definitively identify the origin of these fast Ca²⁺ transient dynamics.

## Discussion

### Imaging performance

Dual-view Bessel two-photon projection microscopy (dv-B2PM) supports 100 volumes per second (VPS) imaging over a 120 × 80 × 42 µm^3^ volume field of view with 0.5 × 0.5 × 0.75 µm^3^ optical voxel resolution and Nyquist sampled with 0.3 × 0.3 × 0.3 µm^3^ digital voxel size. Imaging the same volume at the same resolution by 3D sequential scans would require an effective frame rate of 14 kHz, corresponding to a pixel rate of 1.7 GHz. Such frame or pixel rates have not been applied to 3D synaptic-resolution neuronal imaging because of several key limitations, including the repetition rate of 2PM laser, high readout noise associated with high-speed image acquisition and weak signals from sub-micron structures. Projection imaging compresses 3D information into a slower 2D imaging acquisition and thereby avoids the limitations of the laser repetition rate and high-speed readout noise, but conventional projection approaches lose depth information^15^. Stereoscopy projection imaging was introduced to recover the depth information, but the small angle between two stereoscopic views limits depth resolution and does not reach the synaptic-level^16^. dv-B2PM uses dual orthogonal view projections and maintains sub-micron resolution in all dimensions. Because the two view projections are detected independently by two objective lenses, the system also collects roughly twice as many photons as stereoscopic projection detection and is therefore better equipped to capture subtle activities at the synaptic level. As shown in the raw camera and PMT projection images (Fig. S5c), readout noises in dv-B2PM were relatively low, the major noise source was quantum shot noise in photon signals, and the raw SNR was sufficient for calculating Ca^2+^ signal ΔF/F_0_ in micron-size neural volumes (Fig. S6). If the same 3D activity were instead detected by a hyperphysical 14k FPS 2P light-sheet microscope with similar camera performance, the resulting 140-layer image stack acquired at 100 VPS would accumulate approximately 140 times more image readout noise, making the activity detection impossible.

As an upgrade from Bessel-beam 2P light sheet imaging, dv-B2PM retains highly effective 2P excitation in scattering tissue because of the self-healing property of Bessel beams. The emission image of dv-B2PM does experience resolution degradation in deeper tissue, but at a much slower rate than one-photon light sheet microscope^24^ because of the scatter-resistant excitation and the spatial confinement of 2P excitation. As a result, dv-B2PM maintains synaptic-level resolution in brain tissue to depths of up to 120 µm. This depth limit could be extended in applications focused on somatic activities, where submicron spatial resolution is not needed.

dv-B2PM can be viewed as the equivalent of a dual-angle computed tomography (CT) with 2P emission. With dual orthogonal view projections, dv-B2PM was able to resolve dendritic structures perpendicular to one of the projection planes (Fig. 2b). If single-projection or stereoscopy projection imaging were used, signals along these structures could not be reliably traced and analyzed. dv-B2PM therefore allowed activity tracing and precise measurement of travel distance between major dendrites and soma regardless dendrite orientation. However, as a two-angle CT-like system, some limitations remain. First, dv-B2PM requires sparse neuronal labeling, a common requirement for all neuron imaging, because dense structures would reduce the accuracy of assigning signals to 3D locations. Second, in minor branching dendrites where SNR was low and signal appeared spatially disconnected from major structures, tracing activities was less reliable. These limitations could be mitigated by dual channel-imaging with secondary structure label. Because the instrument can switch freely between light-sheet 3D imaging and dual-projection imaging, it is possible to obtain a true 3D imaging with a structure label to aid analysis and interpretation of fast functional dual-projection data.

### Imaging 3D neuron dynamics in high spatial and temporal resolution

Neuronal information processing relies on a dynamic interplay between dendritic integration, signal propagation toward the soma, and bAPs that travel from the soma back into dendrites. These processes define how neurons transform synaptic inputs into meaningful electrical and biochemical outputs. Although decades of electrophysiological^21,25^ and optical studies^26,27^ have explored these mechanisms, the visualization of spatiotemporal signal flow within 3D dendritic arbors has remained a major challenge. Neuronal signaling occurs across intricate morphologies and over vastly different timescales, and its expression depends strongly on the site, timing, and strength of synaptic activation. Capturing these dynamics therefore requires imaging tools that combine millisecond temporal precision with submicron volumetric resolution—a combination that has been largely unattainable with existing technologies.

Traditional optical imaging approaches often trade spatial coverage for speed. Selected-area imaging captures activity in restricted regions, missing events elsewhere^27^. 2D projection imaging collapses 3D information and introduces structural ambiguity when dendrites overlap or extend perpendicularly to the projection plane^26^. Kilohertz frame rate 2PM, although significantly faster than the standard resonant scanning 2PM, needs to limit the number of layers in 3D scans to achieve a fast volumetric imaging rate^6–8^. Light field imaging sacrifices the spatial resolution to achieve single-shot 3D imaging ability^28^. These limitations obscure key aspects of dendritic computation, including the initiation and propagation of plateau potentials and the spread of Ca²⁺ signals across dendritic branches.

It is worth noting that as a technology that boosts the speed of high-resolution 3D imaging in tissue, the dv-B2PM platform could be combined with genetically encoded voltage indicators^29^ to visualize voltage dynamics and yield direct measurements of neural computation signals. However, current voltage-sensitive indicators generally provide substantially lower signal-to-noise ratios than calcium indicators, particularly in deep brain tissue and at the volumetric imaging speeds required for this study. Calcium imaging, although not a direct measurement of membrane voltage propagation, remains the most practical and valuable approach for investigating the spatiotemporal consequences of dendritic integration in intact neural tissue, especially in high-resolution high-speed imaging studies that are enabled by dv-B2PM.

By integrating dv-B2PM with two-photon glutamate uncaging, we performed visualization of dendritic Ca^2+^ in 3D at 100 Hz refreshing rate. This approach revealed spatially heterogeneous Ca²⁺ transients with nominal frequencies from 5 to 40 Hz that evolved along continuous dendritic pathways, and high-frequency Ca²⁺ transients traveling at greater than millimeter-per-second speeds. Such rapid and localized events over the entire neuron, previously inaccessible with conventional microscopy, likely underlie the mechanisms of dendritic plateau potential generation and signal summation that shape neuronal computation.

Beyond its immediate application to single-cell physiology, dv-B2PM provides a powerful framework for exploring circuit-level integration. Its dual-view design allows volumetric mapping of synaptic activity across interconnected dendritic domains, opening new opportunities to study signal propagation across neurons, plasticity in dendritic ensembles, and spatiotemporal coordination of neuronal microcircuits.

Together, these results establish dv-B2PM as an advanced all-optical platform for dissecting the principles of neuronal integration. By bridging the gap between imaging speed and volumetric resolution, dv-B2PM enables the capture of rapid three-dimensional calcium dynamics that are not readily accessible with conventional volumetric imaging approaches, offering a new window into how the structure and dynamics of dendritic networks give rise to the computational power of the brain.

## Materials and methods

### Animal protocol

All experiments were performed in accordance with protocols approved by Stanford University Animal Care and Use Committee in keeping with the National Institutes of Health’s Guide for the Care and Use of Laboratory Animals.

Thy1-YFP-H (JAX 003782) transgenic mice and C57BL/6 (JAX 000664) were used for imaging. Both were obtained from Jackson Laboratory. Mice are group-housed under a reverse 12 h:12 h light/dark cycle.

To sparsely label neurons with GCaMP8m, viruses (AAV9.CamKII 0.4.Cre.SV40 × CAG-FLEX-GCaMP8m, 1:1000) were delivered to 5–6 week-old C57BL/6 mice brains by stereotaxic injection. Mice were anesthetized with 2% isoflurane and given the analgesic buprenorphine SR (0.5 mg/kg of body weight), and then head-fixed on a stereotaxic frame (Kopf instrument). A glass needle delivered the virus into the motor cortex (M1) of the mouse brain at the coordinate of AP: 1.0 mm, ML: ±1.5 mm from bregma, DV: −0.9 mm from the brain surface. A total of 300 nL virus solution was injected at an infusion rate of 100 nL/min. Following virus injection, the scalp was sutured, and mice were returned to their home cages.

Four weeks after the virus injection, mice were decapitated under isoflurane anesthesia. Brains were extracted and transferred into ice-cold oxygenated (95% CO_2_ and 5% O_2_) artificial cerebrospinal fluid (ACSF, containing (in mM): 125 NaCl, 2.5 KCl, 1.25 NaH_2_PO_4_, 25 NaHCO_3_, 1 MgCl_2_, 2 CaCl_2_ and 15 D(+)-Glucose). 300-μm-thick coronal slices were cut using a vibratome (Leica VT 1200S) and then incubated at 35 °C in ACSF. After 30 min of recovery, the brain slices were maintained at room temperature. During imaging, slices were submerged in an imaging chamber constantly perfused in oxygenated ACSF with 1 mM DNI-Glutamate solution.

Both Thy1-YPF-H and GCaMP8M labeled mouse brain slices were 2P imaged at 920 nm with average power up to 180 mW. The 2P uncaging laser was set at 720 nm, and the power was controlled by the Pockels cell at a desired level. At these laser levels, no significant photobleaching or dendritic structure degrading were noticed during repeated uncaging stimulation and timelapse imaging.

### Optical system of the dual-view Bessel 2P projection microscope

The optical setup of dv-B2PM (Fig. S7) is largely same as our previously published Bessel 2P lightsheet microscope^17^, which is a custom designed up-right microscope with two objective lenses facing each other at 90° angle (OBJ1 and OBJ2 in Fig. 1a and Fig. S7a). These two objectives, respectively, deliver 2P excitation and uncaging lasers to the sample. They also collect fluorescence emissions to form dual-view projection image pairs. The 2P Bessel excitation beam is focused on to the sample through OBJ1 (0.66 NA, 54-10-7, Special Optics), whereas the uncaging beam is focused by OBJ2 (NA 0.8, LUMPLFLN40XW, Olympus). Fluorescence signals collected by OBJ1 are detected by a PMT (H10770PA-40, Hamamatsu), whose output is digitized into a Bessel projection image of the observation volume. Fluorescence signals collected by OBJ2 form a second projection image on the camera (C13440-20CU, Hamamatsu). The system retains its ability to operate at the traditional layer-by-layer 3D light-sheet imaging ability and can be switched between the light-sheet mode and the dv-B2PM mode as needed during experiments.

During imaging, a 920 nm laser beam from a Ti-sapphire laser (Mai Tai HDPS, Spectra-physics) is directed through a Pockels cell (Model 350-80, Conoptics), which controls the excitation power and exposure timing. The beam then passes through a beam expander and an axicon lens (AX251-B, Thorlabs) and is shaped into a Bessel beam. The Bessel beam is projected onto a resonant galvanometer scanner (CRS 8 kHz, Cambridge Technology), which scans the beam in the x-direction at 8 kHz. A close-loop galvanometer scanner (GVS001, Thorlabs) follows and scans the beam in z-direction. The scanning Bessel excitation beam is then relayed onto the back aperture of OBJ1 and focused into a thin Bessel excitation beam with an extended focal length of 36.8 μm (FWHM) and a width of 0.75 μm (FWHM). The excitation beam scans across the imaging volume at a speed of 50 Hz, which supports 100 VPS projection imaging (See Fig. S8a for imaging control diagram). The fluorescence signals collected by OBJ1 are detected by the PMT, forming y-axis projection images at a framerate of 100 Hz. On the OBJ2 side, an ETL (EL-10-30-CI-VIS-LD, Optotune) is placed at the conjugated plane of the back focal plane of OBJ2. The ETL is driven by a 50 Hz sinusoidal current with 140 mA peak-to-peak amplitude, which moves the camera focal depth in the z-direction over a 42 μm range in synchronization with the driving waveform of the z-direction galvanometer scanner (ZG) (Fig. S8b).

The camera is set in the global exposure mode and captures projection images at a 100 Hz exposure rate. The exposure timing is precisely controlled by the Pockels cell, which turns on and off the excitation laser with sub-millisecond timing accuracy, better than the electric exposure control of the camera (Fig. S8b). The camera and PMT capture dual-view projections simultaneously during z-scanning. In a previous study, we had developed a method for precise matching between the scanning laser beam and ETL-driven focusing dynamically during fast volume scans^17^. The method allowed the Bessel excitation beam dynamically tracking the camera scan in the z-direction during an exposure and creating a z-projection image of the entire volume during the exposure.

### Projection data acquisition and processing

The camera and PMT captured the dual-view projection data simultaneously. With the 8 kHz resonant scanner driving the x-scan, each volume contained 140 layers of bidirectional scan stacked in the z-direction at 0.3 μm step size. Camera images were read out at 400 × 400 frames with 0.3 μm pixel size, yielding a digital voxel size of 0.3 × 0.3 × 0.3 µm. The region of interest size in the camera projection image is typically 400 × 300 pixels, resulting in a x–y lateral field of view of 120 μm × 80 μm (Fig. S5a). The PMT captured x–z plane projection images at 20 MHz sampling rate. The raw data stream from the PMT was first organized into x–z plane projection image frames, corrected for nonlinearities in the resonant scanner, interpolated to an even x–z pixel grid, and finally trimmed to match with the x-dimension field of view size in the camera image (Fig. S5b). This process resulted in a dual view time-lapse image set at 100 Hz (Fig. S5c).

Both the camera and the PMT projection movies were corrected for sample drifting. All the data processing was carried out in MATLAB.

### Two-photon glutamate uncaging

We used a second Ti-sapphire laser (Mai Tai HP, Spectra-physics) for uncaging glutamate at 720 nm. The uncaging beam focus was independently controlled by a cell modulator (Model 350-80, Conoptics), a close-loop galvo scanner set (x–y galvo set, GVS001, Thorlabs), and a near infrared (NIR) ETL (EL-10-30-CI-NIR-LD, Optotune). We verified that the focused uncaging beam provides submicron resolution stimulation by measuring the transverse and axial profile of the uncaging focus. 3D focus measurements showed near diffraction-limited sizes across the field-of-view (Fig. S9). The positioning of the uncaging focus was calibrated by imaging the focus in the camera. The targeting precision was confirmed with a single spine photobleaching test in a Thy1-YFP mouse brain slice (Fig. S10), during which a single spine head was selected after a first round of imaging and subjected an intense exposure to the focused uncaging beam. A second round of imaging showed that the spin was bleached whereas fluorescence signals of surrounding structures remained the same.

### Imaging 3D structure and Ca^2+^ activity

To validate 3D structures seen through dv-B2PM imaging, we performed both dv-B2PM and 3D-stack light-sheet imaging of the same neuronal structure (Fig. S11). Projection images from the camera and PMT matched well with pseudo projection images calculated with the true 3D light-sheet image stack.

To image Ca^2+^ activities under two-photon glutamate uncaging, a section of GCaMP8m-labeled mouse brain slice was anchored by a slice harp in the imaging chamber. Under the guidance of dv-B2PM, we first selected a region of interest and a potential uncaging location, typically 50–100 µm deep from the slice surface, as superficial neurons are less likely to retain normal dendritic structures, and hence the physiological responses to synaptic stimulations. We then performed 3D-stack light-sheet imaging in a small volume near the potential uncaging location to map dendritic structure in the vicinity and set the precise 3D location of targeted uncaging, typically near a dendrite spine. Testing of uncaging under small volume 3D light sheet imaging was then conducted to verify the effectiveness of uncaging. In the final step, the image mode was switched back to the dv-B2PM mode and 3D activity induced by two-photon glutamate uncaging were images at 100 VPS with an extended volume size trying to capture the whole cell. The maximal depth of the image volume is typically limited to 120 µm because image resolution degradation in deep tissue may exceed the sub-micron resolution needed for this study. The experiment generated two Ca^2+^ activity data sets, a slow small volume 3D-stack light-sheet image set and a 100 VPS large volume image set from dv-B2PM (Fig. S12). Between the two image modes, dv-B2PM delivered a much faster volume rate within a larger image volume, allowing more comprehensive study of neural activities.

During Ca^2+^ activity imaging, the typical uncaging onset timing was 2–5 s after the imaging began. The 720 nm uncaging laser pulse was set to 50 Hz at a 50% duty cycle. The uncaging stimulation lasted a total 20 ms, during which 10 pulse cycles were applied to the stimulation location. A typical imaging session lasted 30 s. Multiple imaging sessions of the same location were separated by at least 4-minute intervals.

### Extracting Ca^2+^ signals in neuron structure sections

To analyze 3D Ca^2+^ activities, structures that exhibit activities in both projection views were traced in both the camera image and the PMT image through an interactive MATLAB program. The program first used both locations of active Ca^2+^ signals together with static “landmark” Ca^2+^ features to align the two projection images along the x-axis (Fig. S5c). It then traced between user defined dendrite start and end points, identified 3D locations of maximal Ca^2+^ activity along the dendrite in the conjoint view of the camera (x–y) and the PMT (x–z), and generated an array of continuous 3D coordinates of the dendrite. These coordinates allowed dendrite signals being extracted from both views are single pixel levels (Fig. S6a). The program also determined the soma coordinate region based on user defined boundaries.

To analyze signals along the dendrite, the program automatically created 3D cylinder segments of dendrite structures (3 µm in length along dendrites, 0.6 µm in diameter and 1.5 µm in incremental steps) and extracted signal time-traces of all segments from the camera and the PMT projection images (Fig. S6b). Each pair of signal time-varying traces of the same section, from the camera and the PMT respectively, are averaged into a single merged trace (Fig. S6c). The resulting Ca^2+^ ΔF/F_0_ signal typically have peaks SNR between 5 and 20 in most 3-µm-long dendrite segments (Fig. S6d). Soma signals were calculated by averaging all voxels within the soma volume, and their peak SNR exceeded 30. All Ca^2+^ ΔF/F_0_ signal traces were sent to spatial-temporal analysis without filtering or denoising, as these digital signal enhancement methods could affect the short time-scale spatial-temporal analysis results.

### Spatial-temporal analysis

In long-timescale analysis, calcium signal traces were fitted with a multi-peak model of exponentially modified Gaussian peaks. The fitting program automatically adjusts the number of peaks based on fitting residuals analysis. The program outputs timing of all Ca^2+^ signal peaks, peak amplitudes and duration factors. In short-timescale analysis, 2D continuous wavelet analysis were performed on calcium signals along the dendrite. The wavelet analysis used the 2D third-order Gaussian wavelet kernel that consists of a single peak-to-valley transition in the 2D spatial-temporal domain, which was designed to analyze short individual fluctuations. The wavelet kernel was scaled in time to four kernel functions centered at nominal frequencies of 5, 10, 20 and 40 Hz respectively (Fig. S13), targeting fluctuations at four different timescales. All analysis were performed in MATLAB.

## Supplementary information

Supplementary Information

## Data Availability

Image data and scripts used to analyze the data can be downloaded at.https://zenodo.org/records/17887796.
