# Supplementary material for: Two-photon 3D imaging of optically stimulated neural activity at 100 Hz
Source: Light Sci Appl. 2026 Jul 3;15:303. doi: 10.1038/s41377-026-02395-2 (PMC13328388; doi:10.1038/s41377-026-02395-2)
Supplement: Supplementary file 1 — Supplementary Information [file 41377_2026_2395_MOESM1_ESM.pdf]

Supplementary Information for

Two-photon 3D imaging of optically stimulated neural activity at 100Hz

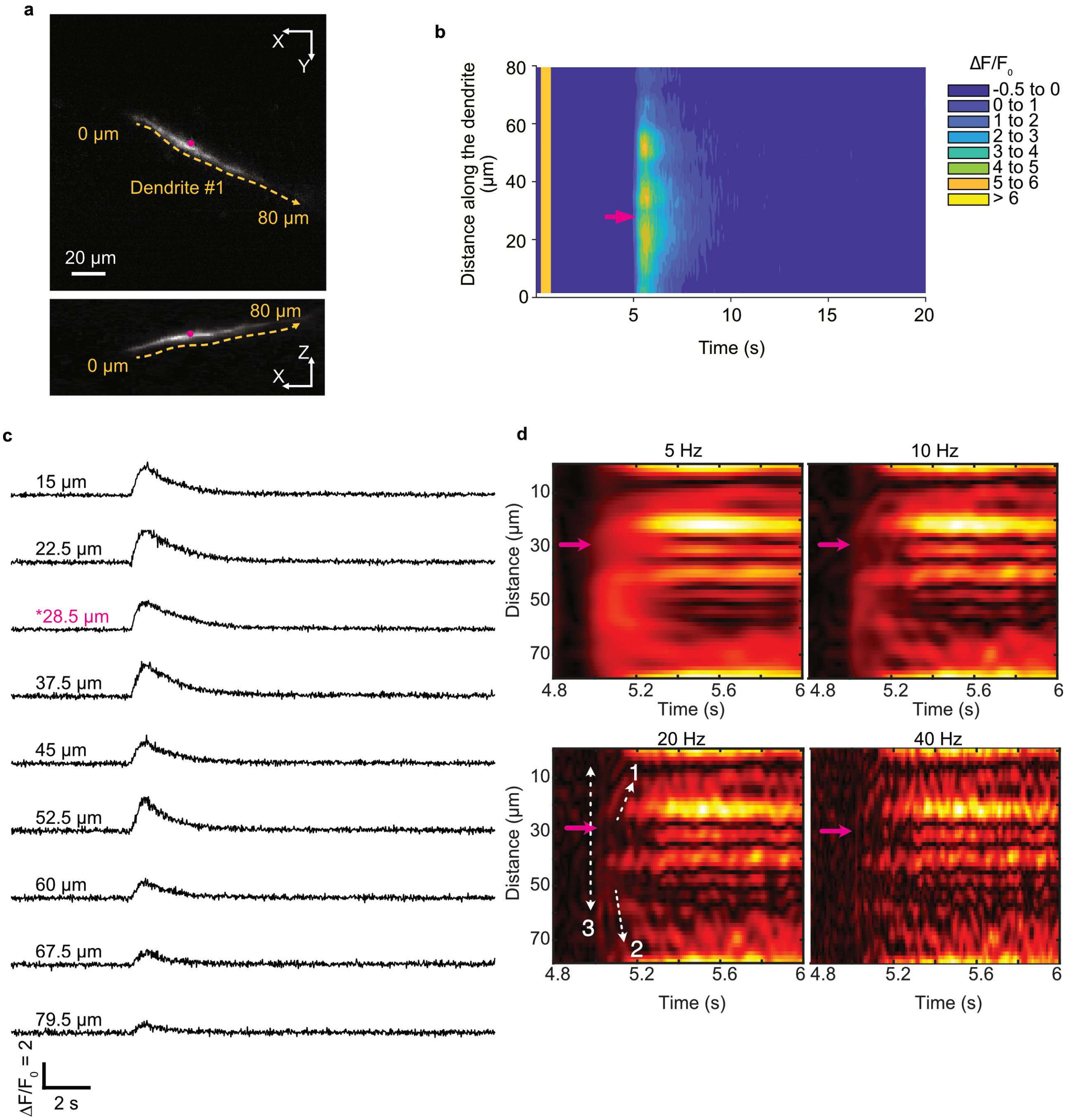

Fig. S1 Localized calcium activity after under-threshold uncaging stimulation, captured by dv-B2PM. Magenta markers indicates the uncaging point. The uncaging onset time was at 5 second. The uncaging beam power was 60 mW.

(a) Top: Camera projection image. Bottom: PMT projection image. Image intensities represent the calcium activity changes (ΔF) within the 1-second-long time window after the uncaging onset. The imaging volume rate was 100 Hz. The magenta marker indicates the uncaging point.

(b) Calcium activity spatiotemporal heat map of the 80-μm-long dendrite section. The uncaging was activated next to the 28.5 μm location at 5 second.

(c) Calcium activity time traces at selected locations along the dendrite section.

(d) Wavelet analysis results. Highlighted area in heat maps indicate fluctuations that persisted in the space-time. These heat maps are modular amplitudes of 2D continues wavelet analysis of calcium signals. The analysis used the third order 2D gaussian wavelet function at centered at 5, 10, 20 and 40 Hz (Fig. S13). At 20 Hz, short local propagations at 175 $\mu m \cdot s^{-1}$ (#1 arrow) and 471 $\mu m \cdot s^{-1}$ (#2 arrow) away from the uncaging point are seen. At 20 and 40 Hz, analysis shows a leading-edge propagation (#3) traveling across the dendrite faster than the speed of acquisition.

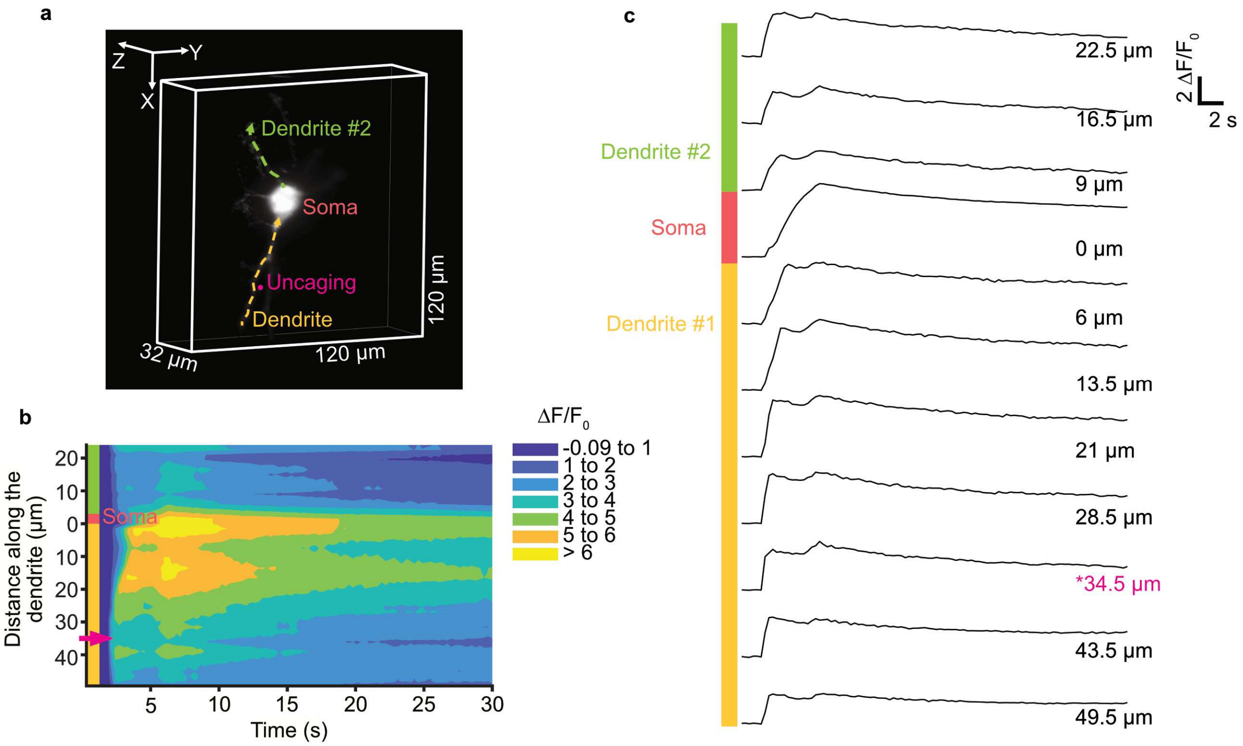

Fig. S2 Extended calcium activities in a neuron after over-threshold uncaging stimulation, captured by 3D light-sheet stack scan. The volume size is 120 × 80 × 32 μm, and was captured with 1 μm z-step stack scan. The volume refreshing rate is 3.3 Hz. The uncaging onset time was at 2 second. The uncaging beam power was 60 mW.

(a) Maximum intensity projection of the image stack. Image intensities represent the average calcium activity changes (ΔF) within the 1-second-long time window after the uncaging onset.

(b) Calcium activities spatiotemporal heat map along the path from dendrite#1 to soma and then to dendrite #2 as shown in (a).

(c) Calcium activity time traces at selected locations along the path.

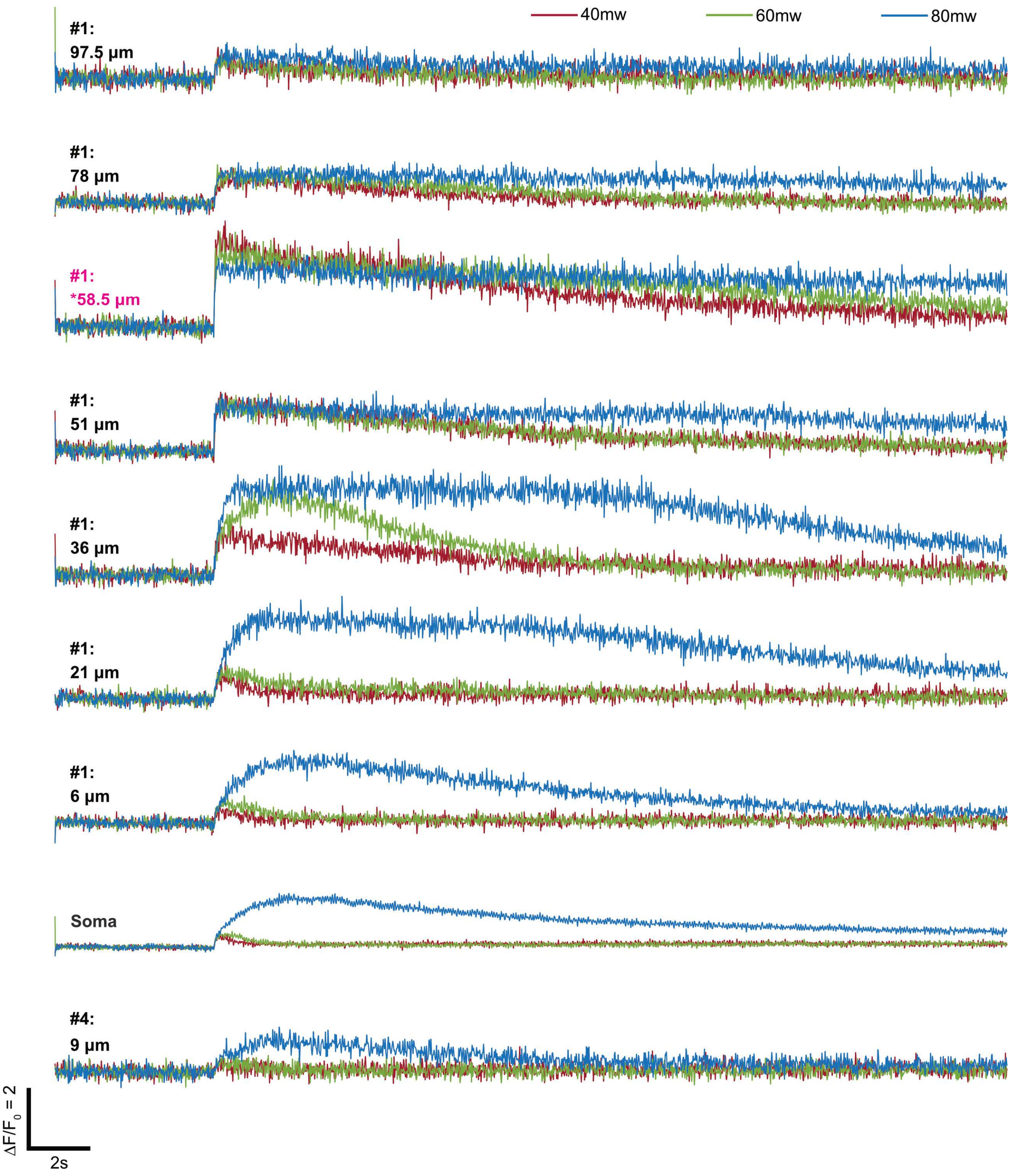

Fig. S3 Calcium time traces at selected locations along the dendrite #1-to-soma-to-dendrite #2 path in Fig. 3.

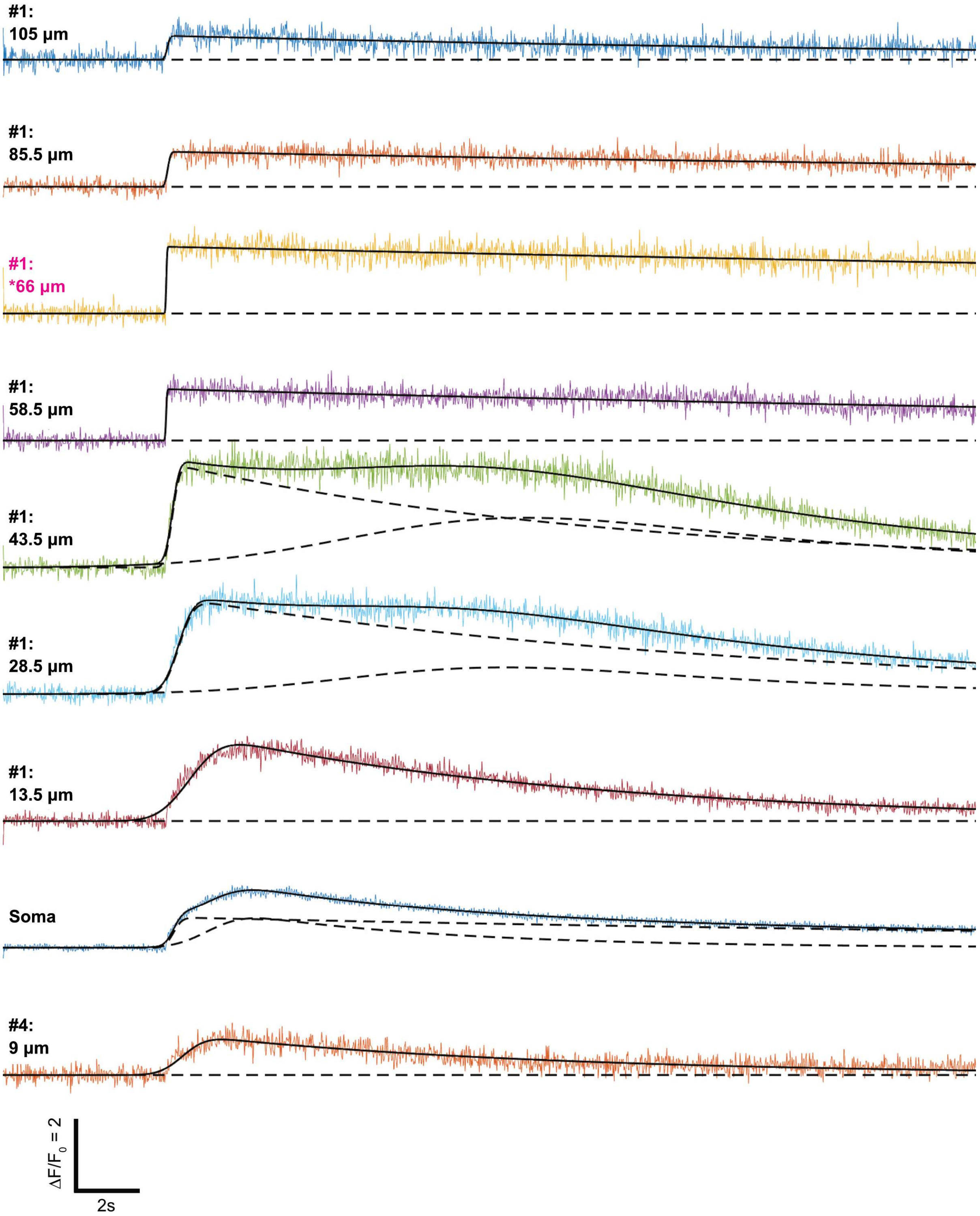

Fig. S4 Long timescale calcium signal time-space analysis. Calcium signals were fitted with a double-peak model (solid lines). Individual peaks of the total fitting curve are plotted in dashed

lines. At locations without distinct secondary peaks, the fitting converges to the single peak model and assigns a zero amplitude on the second peak. Timing of early (or single) peaks and second peak are plotted in Fig. 3c.

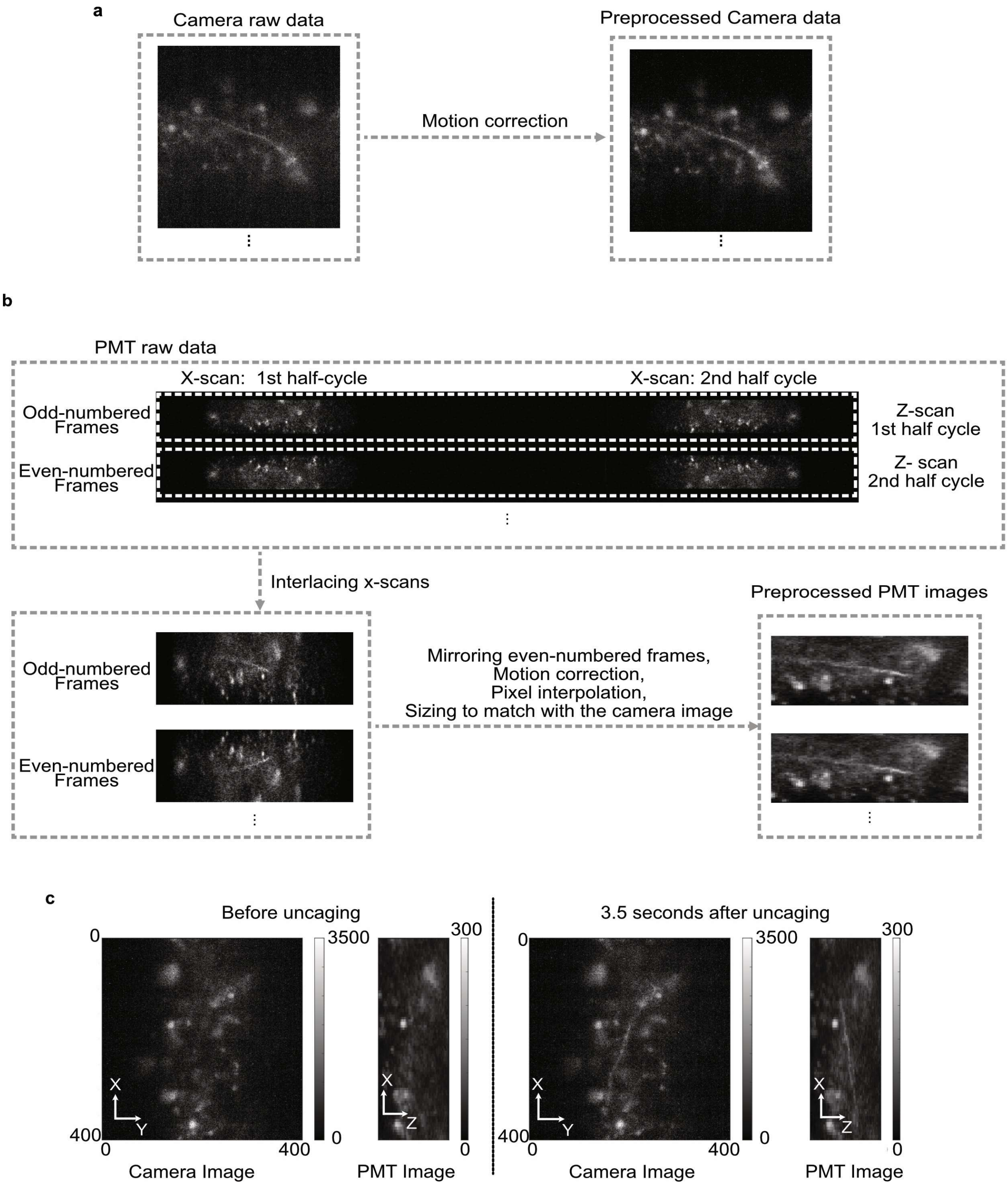

Fig. S5 Data pre-processing procedures of dv-B2PM. All the data processing procedures are conducted using MATLAB.

(a) Camera data pre-processing. All the projection images captured by the camera were corrected

from minor sample drifting. The pixel size of the camera image is 0.3 μm.

(b) PMT data pre-processing. The raw resonant scanning PMT data steam was first organized into a projection image sequence. Then, the PMT projection image sequence underwent motion correction. Finally, the pixel size of the PMT projection images was interpolated into an isotropic 0.3 μm pixel size grid.

(c) Representative projection images from the camera and the PMT after the data pre-processing. Two projection views were alignment in x-direction by both location of active Ca^{2+} signals and highly localized "landmark" static Ca^{2+} signals. The typical readout noise standard deviation, measured in dark areas in images, is 90 units in the camera, 7 in the PMT. Both are smaller than 10% of typical activity signals, which are greater than 1000 units in the camera and 100 units in the PMT.

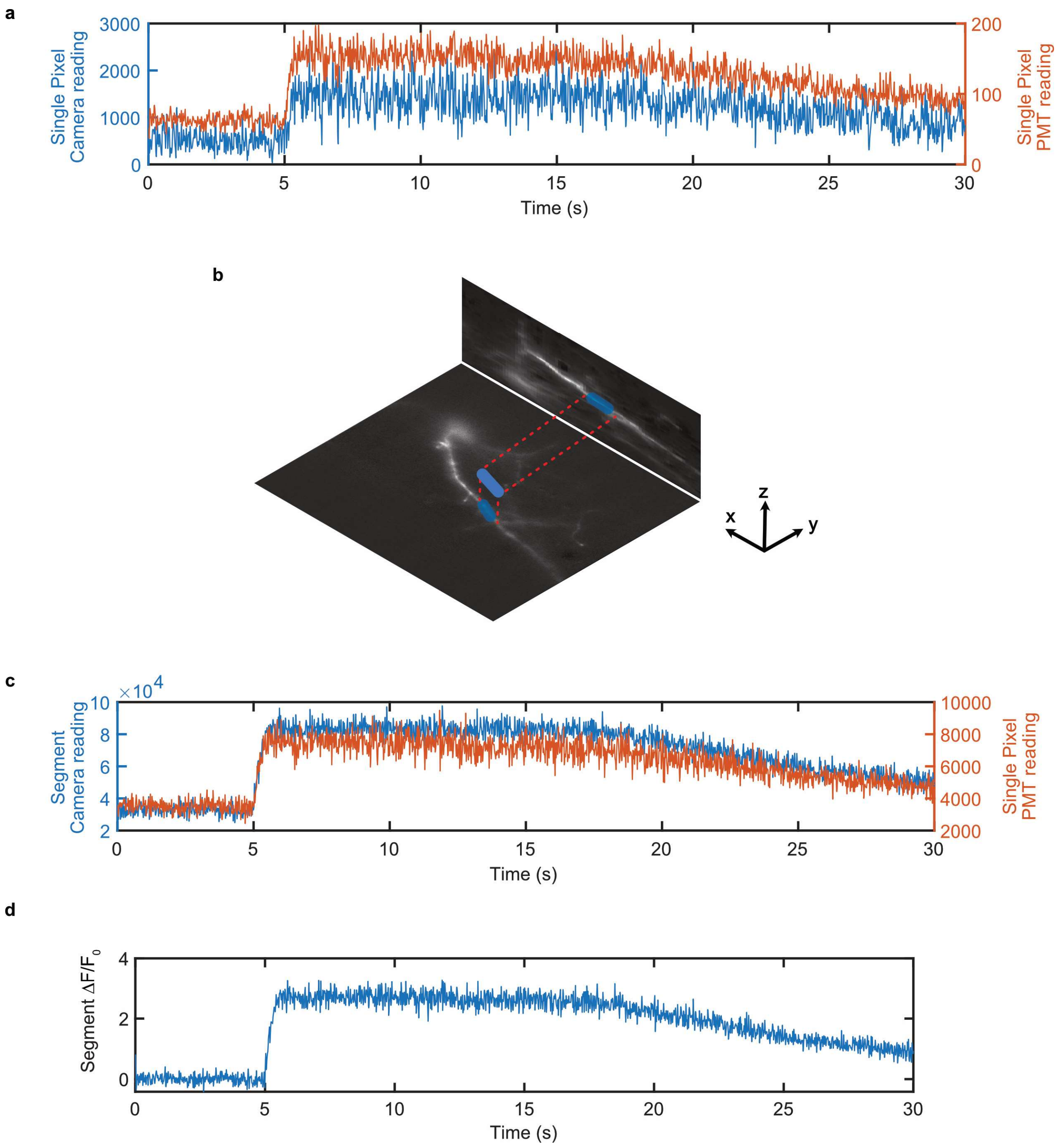

Fig. S6 Analyzing Ca^{2+} signal along dendrite structures

(a) Representative single voxel Ca^{2+} signals from the corresponding pixel in the camera and PMT image respectively. Two time-traces show good agreements.

(b) Integrating signals within a 3D cylinder segment of the dendrite. Pixels fall within the projection of the segment (blue shadows in two projection planes) were averaged in the camera

plane (x-y) and the PMT plane (x-z) respectively. The cylinder and its shadow areas in the figure are larger than the actual size for illustration purposes. The cylinder size used for this work is 3 μm long and 0.6 μm in radius.

(c) Representative segment Ca^{2+} signals from the corresponding projection area in the camera and PMT image respectively.

(d) Signals from two planes were normalized and weighted averaged by

$$F(t) = wF_{Cam}(t) + (1-w)F_{PMT}(t)\frac{\overline{F_{Cam}(t)}}{\overline{F_{PMT}(t)}}$$

where the weight coefficient is calculated based on the baseline noise

$$w = \frac{std[F_{PMT}(t < t0)]}{std[F_{Cam}(t < t0)] + std[F_{PMT}(t < t0)]}$$

$\Delta F/F_0$ were calculated from the merged F(t). The resulting $\Delta F/F_0$ has a typical baseline noise of 0.2 and SNR between 5-20 in most dendrite segments.

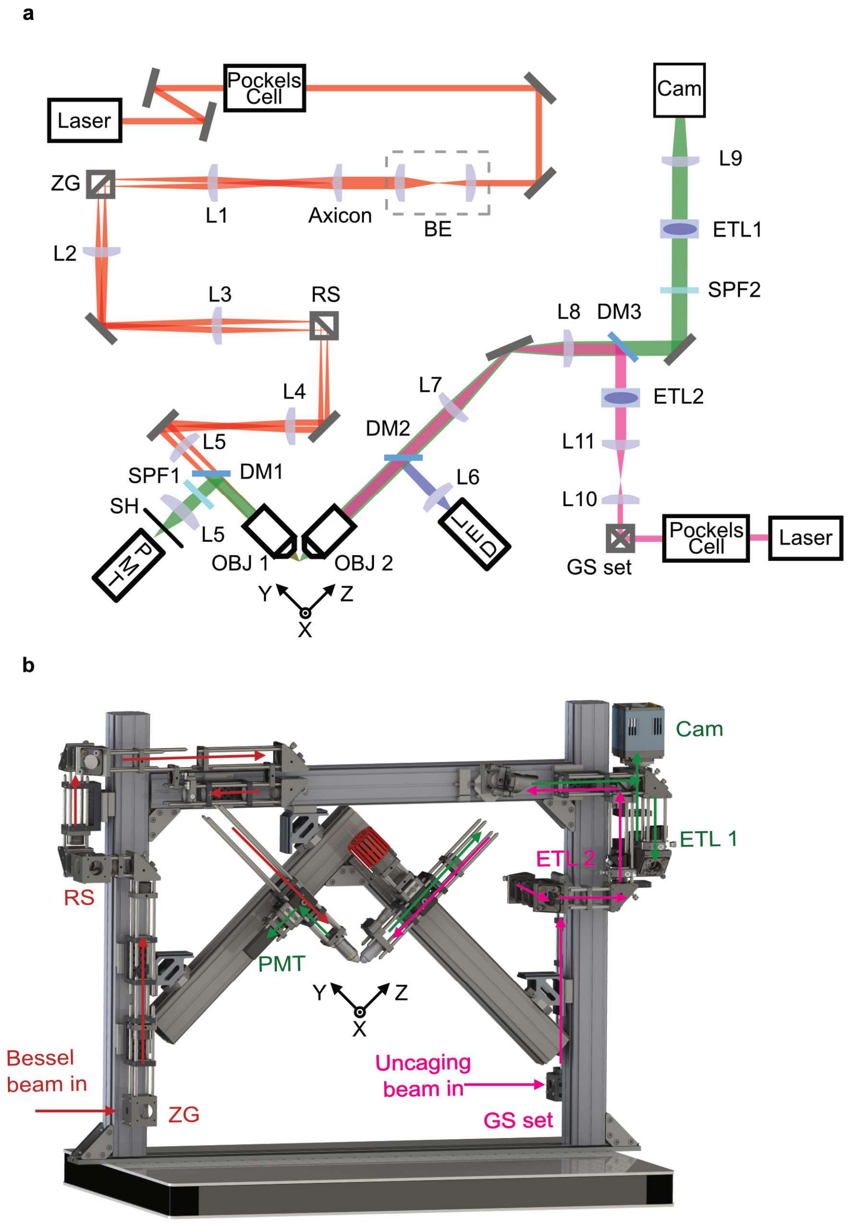

Fig. S7 Optical layout of dual-view Bessel 2P projection microscope. The optical trains for imaging scanning, fluorescence collection and uncaging are marked with red, green and magenta respectively. BE, beam expander; L, lens; ZG, Z galvo scanner; RS, resonant scanner; DM, dichroic mirror; SPF, short pass filter; SH, shutter; PMT, photon multiplier tube; OBJ, objective lens; ETL, electrically tunable lens; GS set, galvo scanner set; Cam, camera.

(a) Optical schematic of dv-B2PM.

(b) A CAD-rendering of the dual-view Bessel 2P projection microscope.

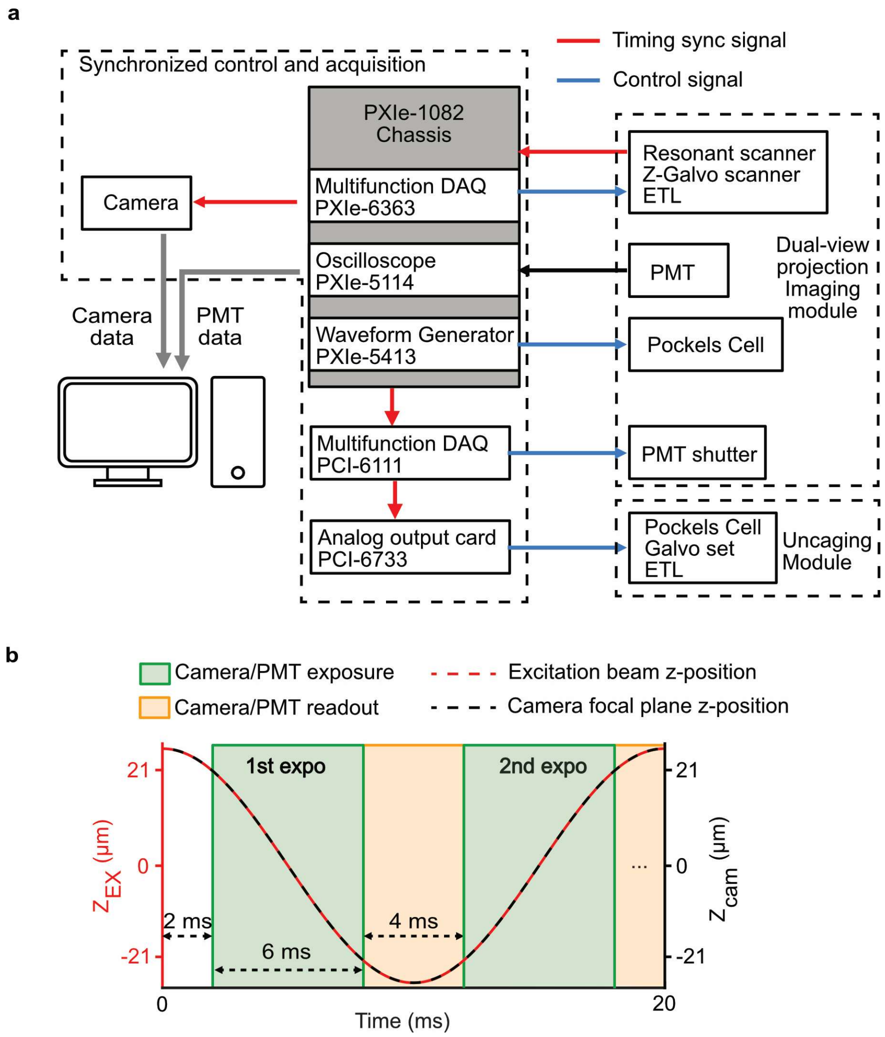

Fig. S8 Imaging control of dv-B2PM system

(a) Data acquisition and instrument control of dv-B2PM system. A synchronization signal from the resonant scanner works as a trigger to synchronize all the imaging and uncaging processes. Key components and detectors are controlled by the PXIe-1082 chassis (National instruments), DAQ/AO cards (National instruments), and a host computer. The acquisition software was developed in LabVIEW.

(b) Acquisition timing diagram of dv-B2PM. The camera focal plane is tuned by a 50 Hz sinusoidal wave driven ETL1 in z-direction. The Bessel excitation beam is tightly synchronized with the movement of the camera focal plane, scanning through the volume in

the z-direction. The camera and PMT take projection images bidirectionally at a speed of 100 frames per second. Each exposure lasts 6 ms. The 4-ms time periods of non-linear z-scanning are used for the data readout.

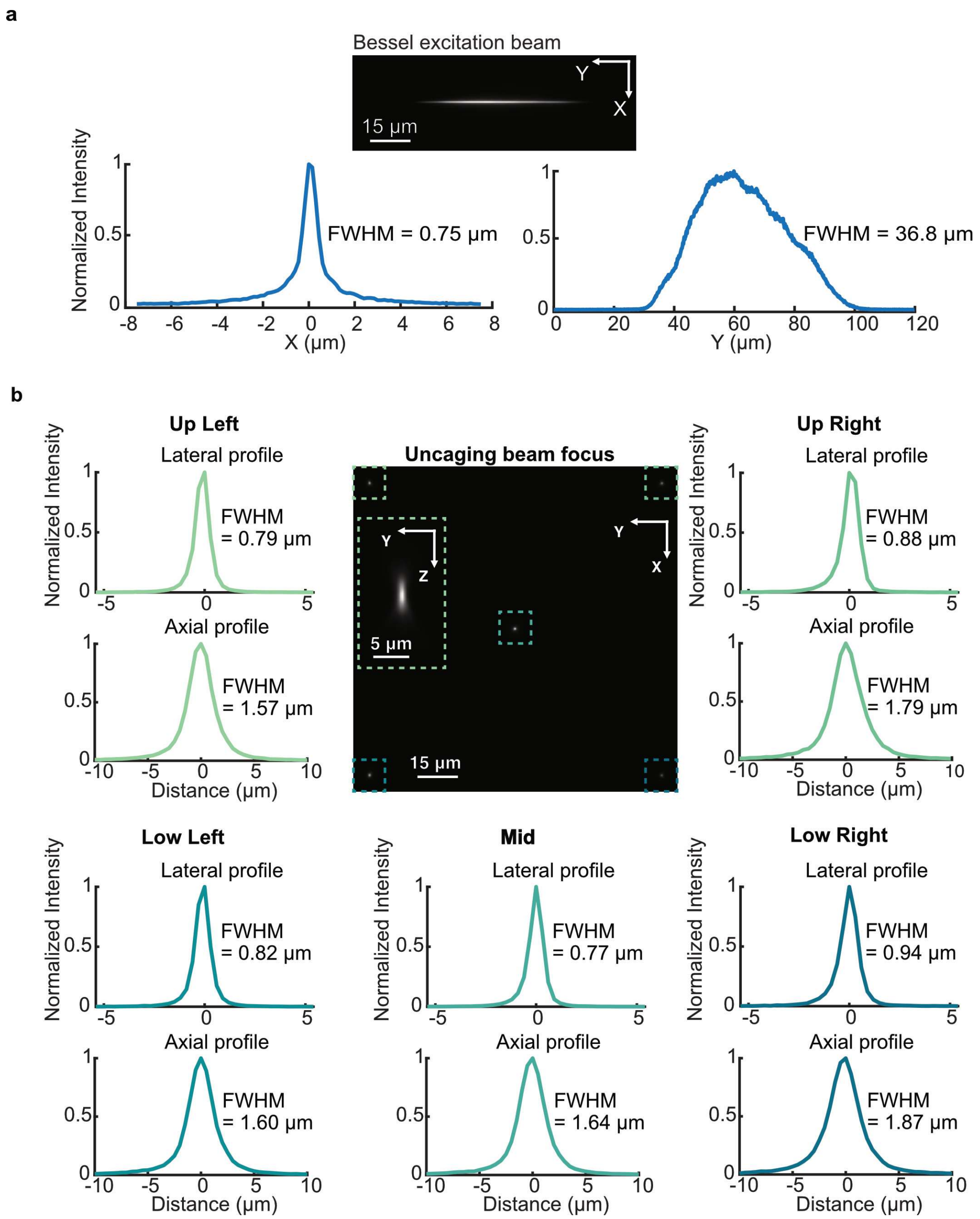

Fig. S9 Characterization of the focused Bessel excitation beam and the 2P uncaging focus.

(a) Top: a cross-section image of the focused Bessel excitation beam, taken by focusing a stationary Bessel beam in a dye solution and capturing the x-y plane fluorescence image with the camera. Bottom left: Cross section of the beam at x direction, showing a FHMW width of is 0.75 μm. Bottom right: Cross section along the length of the beam (y-direction), which has

a FWHM of 37 µm and supports approximately 80 µm wide field of view in the y direction.

(b) Characteristics of 2P uncaging beam focus over the imaging field-of-view (FOV). The uncaging beam was directed to the middle and four corners of the imaging FOV successively. Five point spread functions (PSFs) of the uncaging foci were captured using 3D light-sheet imaging. all PSFs have consistent FWHMs in 3D that are close to theoretical predictions based on ideal lens diffraction.

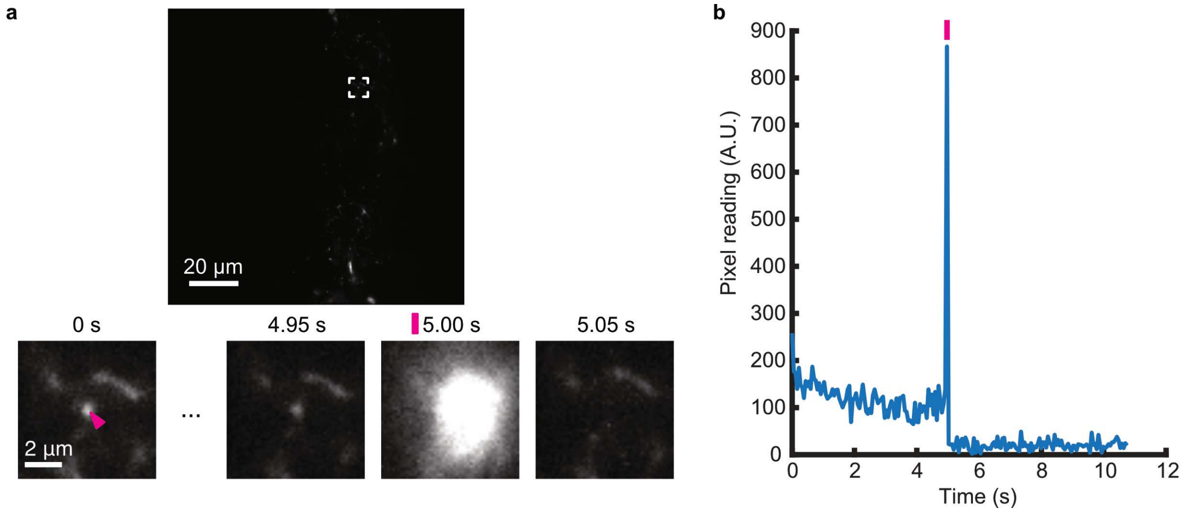

Fig. S10 Verifying the spatial accuracy of uncaging by photobleaching test of a dendrite spine in Thy1-YFP mouse brain slice using the uncaging laser focus.

(a) Selected light sheet images during the bleaching test. A single spine was exposed to a 1-ms-long uncaging laser pulse with a 100 mw power. The spine was bleached without affecting the surrounding fluorescence signals, demonstrating the uncaging procedure was precise and highly localized. Top: Full view of the light sheet image. Bottom: Selected time-laps frames of the region of interest (dashed box in the full view), showing the bleaching of a single spin (pink arrow) by the uncaging focus at 5 second.

(b) Fluorescence signal trace of the targeted spine. The fluorescence signal of the targeted spine dropped to the background level after being exposed to the 720 nm laser pulse.

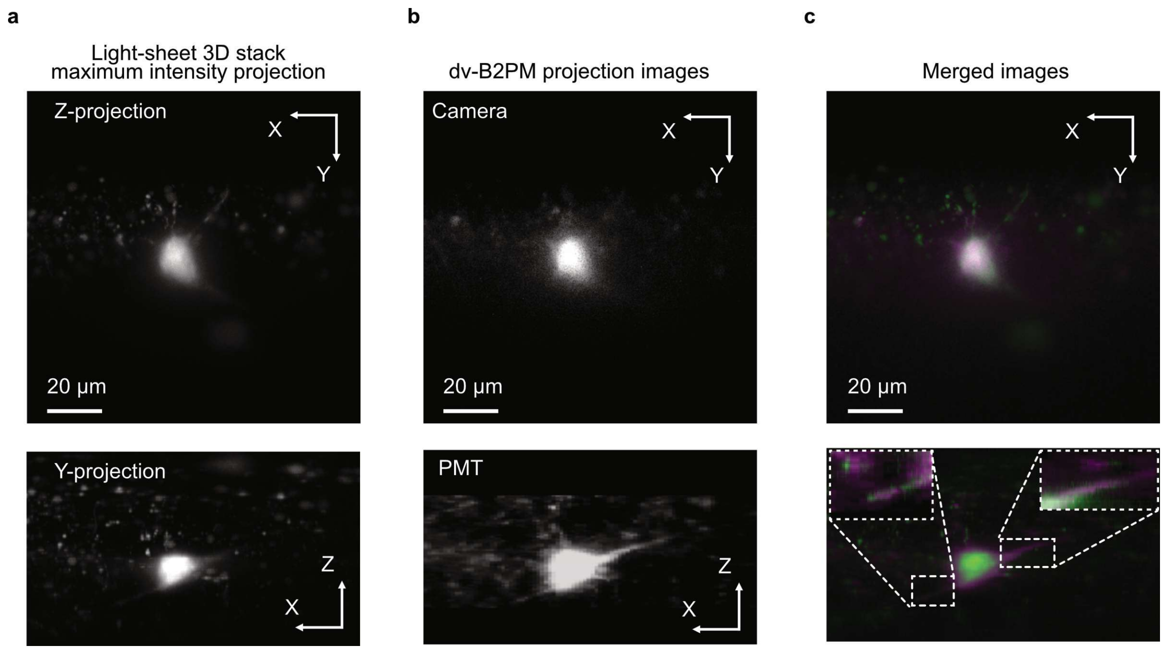

Fig. S11 Comparing 3D light-sheet stack images and dv-B2PM images of a neuron in a motor cortex (M1) Thy1-YFP mouse brain slice.

(a) Maximum intensity projection (MIP) images of the 3D light-sheet image dataset, showing a neuron centered in the imaged volume. The image set contains 70 light-sheet layers at a z-step step of 1 μm.

(b) Projection images of the same neuron captured by the Camera and PMT using dv-B2PM. The imaged volume covered 120 μm × 80 μm × 42 μm.

(c) Merging of the pseudo projection images calculated from light sheet 3D images and experimentally acquired dv-B2PM projection images. The merge shows good agreements between two imagine modes.

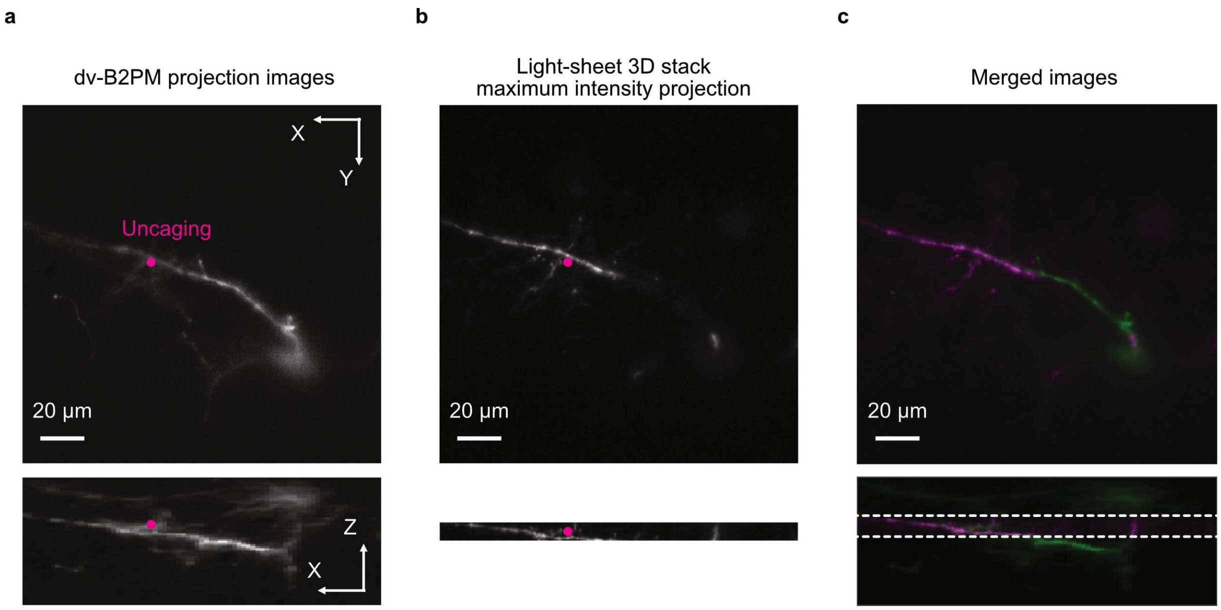

Fig. S12 Comparing dv-B2PM imaging and 3D light-sheet imaging of glutamate uncaging-induced calcium activities in a neuron.

(a) Calcium activity projection images captured by dv-B2PM. Image intensities represent maximum projection of the calcium activity changes (ΔF) within the 1-second-long time window after the uncaging onset. The imaged volume was 120 µm × 80 µm × 42 µm. The imaging volume rate was 100 Hz.

(b) Pseudo projection images of calcium activity, render from the light-sheet 3D image stack on the same neuron using the same uncaging protocol. The imaged volume was 120 µm × 80 µm × 6 µm with 0.5 µm z-step. The camera framerate was 100 Hz, and the imaging volume rate was 8.3 Hz.

(c) Merging of the dv-B2PM projection images and pseudo projection images calculated from 3D light-sheet image stacks. dv-B2PM delivered a much faster volume rate and a larger imaging volume.

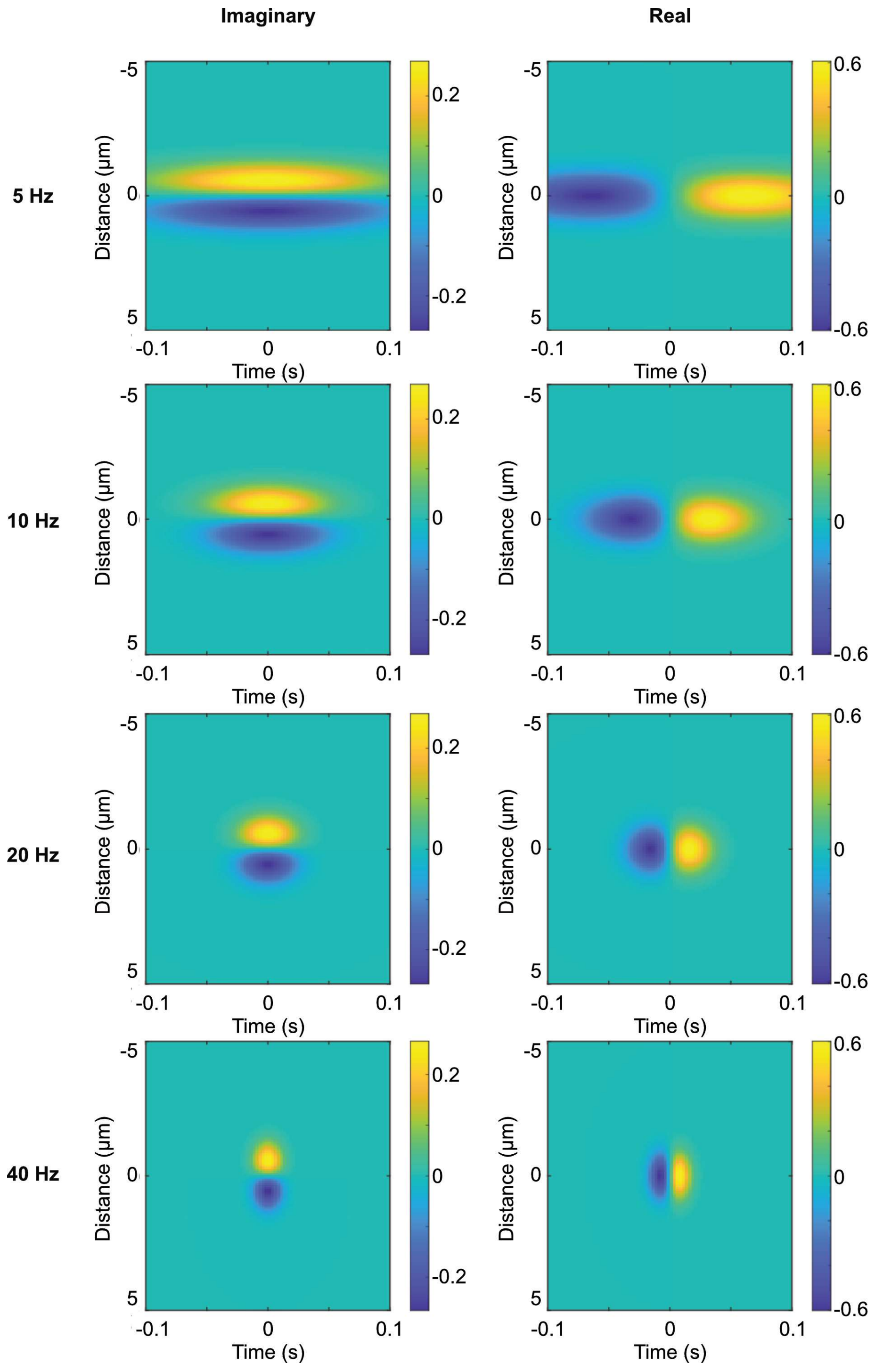

Fig. S13 2D Gaussian wavelets used for 2D spatiotemporal wavelet analysis of Ca^{2+} signals. Four wavelets centered at 5, 10, 20 and 40 Hz were used. The nominal spatial resolution of all wavelets were kept the same at 5 µm.

No.	Direction	Distance (μm)	Duration (ms)	Speed (mms)	Freq (5-40Hz)	Stim (mW)
**1**	Soma	-59	40	1.5	Low	All
**2**	Distal	15	30	0.5	Low	All
**3,4**	Soma	-17	670	0.025	Low	40,60
**5**	Soma	-26	100	0.26	Mid	60
**6**	Both	±20	<10*	>2	High	60,80
**7**	Soma	-42	250	0.17	Mid	80

Table. S1 Spatial-temporal characteristic of calcium transient traces in apical dendrite observed after uncaging. * The time delay is shorter than the projection acquisition time.
